# Bilateral sectioning of the anterior ethmoidal nerves does not eliminate the diving response in voluntarily diving rats

**DOI:** 10.1002/phy2.141

**Published:** 2013-11-07

**Authors:** Jill S Chotiyanonta, Karyn M DiNovo, Paul F McCulloch

**Affiliations:** Department of Physiology, Midwestern UniversityDowners Grove, Illinois

**Keywords:** Anterior ethmoidal nerve, diving response, nasopharyngeal response, neuronal plasticity, rats

## Abstract

The diving response is characterized by bradycardia, apnea, and increased peripheral resistance. This reflex response is initiated by immersing the nose in water. Because the anterior ethmoidal nerve (AEN) innervates the nose, our hypothesis was that intact AENs are essential for initiating the diving response in voluntarily diving rats. Heart rate (HR) and arterial blood pressure (BPa) were monitored using implanted biotransmitters. Sprague-Dawley rats were trained to voluntarily swim 5 m underwater. During diving, HR decreased from 480 ± 15 to 99 ± 5 bpm and BPa increased from 136 ± 2 to 187 ± 3 mmHg. Experimental rats (*N* = 9) then received bilateral AEN sectioning, while Sham rats (*N* = 8) did not. During diving in Experimental rats 7 days after AEN surgery, HR decreased from 478 ± 13 to 76 ± 4 bpm and BPa increased from 134 ± 3 to 186 ± 4 mmHg. Responses were similar in Sham rats. Then, during nasal stimulation with ammonia vapors in urethane-anesthetized Experimental rats, HR decreased from 368 ± 7 to 83 ± 4 bpm, and BPa increased from 126 ± 7 to 175 ± 4 mmHg. Responses were similar in Sham rats. Thus, 1 week after being sectioned the AENs are not essential for initiating a full cardiorespiratory response during both voluntary diving and nasal stimulation. We conclude that other nerve(s) innervating the nose are able to provide an afferent signal sufficient to initiate the diving response, although neuronal plasticity within the medullary dorsal horn may be necessary for this to occur.

## Introduction

The diving response is triggered when animals dive underwater (Butler and Jones 1997). This reflex response includes apnea, an immediate and intense decrease in heart rate (HR), and an increase in arterial blood pressures (BPa) caused by peripheral vasoconstriction (McCulloch et al. 2010). These dynamic cardiovascular adjustments allow an animal to efficiently use on-board oxygen stores while under water and unable to breathe (Butler and Jones 1997). It is currently thought that the neural circuitry of the diving response starts with stimulation of the nose and upper respiratory tract (Butler and Jones 1997; McCulloch 2012), particularly through activation of the anterior ethmoidal nerve (AEN) that innervates the external nares and nasal passages (Greene 1963). The central axon terminals of the AEN project to the spinal trigeminal nucleus caudalis (Panneton et al. 2006; Hollandsworth et al. 2009), which is also known as the medullary dorsal horn (MDH). Secondary neurons within the MDH appear to be an important part of the afferent pathway involved in integrating the signal that produces the cardiorespiratory changes observed during diving (McCulloch and Panneton 1997; McCulloch 2005; Panneton et al. 2006).

A diving-like response can be initiated in anesthetized animals by several methods other than actual diving. When the nasal mucosa is stimulated by water (McCulloch and West 1992; McCulloch and Panneton 1997), chemical irritants like ammonia vapors (Panneton 1991b; Rybka and McCulloch 2006) or 100% carbon dioxide (Yavari et al. 1996), there is initiation of a nasopharyngeal reflex that appears to be qualitatively and quantitatively similar to the diving response. This diving-like response is also produced when the AEN itself is electrically stimulated (McCulloch et al. 1999). Past studies have investigated the nasopharyngeal reflex by applying local anesthetic to nasal passages (McCulloch et al. 1995) or MDH (Panneton 1991b), applying glutamate receptor antagonists within MDH (McCulloch et al. 1995; Panneton and Yavari 1995), or even acutely cutting the AENs bilaterally (McCulloch et al. 1995; Rybka and McCulloch 2006). These methods all either eliminated or greatly attenuated the nasopharyngeal response to nasal stimulation, suggesting that the AEN is important in initiating the diving-like response in anesthetized animals. This has led to the supposition that the AEN is important in initiating the diving response in conscious voluntarily diving animals.

However, to date, no study has examined the cardiovascular responses in voluntarily diving conscious rats without intact AENs. The present project used conscious rats trained to swim underwater. The cardiovascular responses during voluntary diving, monitored using implanted BPa biotelemetric transmitters, were recorded before and after bilateral sectioning of the AENs. The hypothesis of this study was that intact AENs are essential for initiating the diving response in voluntarily diving rats.

## Materials and Methods

### Training

All study procedures were approved by the Midwestern University Institutional Animal Care and Use Committee (IACUC). Three-week-old male Sprague-Dawley rats were purchased from a commercial vendor (Harlan, Indianapolis, IN). Rats were caged in pairs and were housed in accordance with National Institutes of Health (NIH) guidelines for the care and use of laboratory animals. Food and water were available ad libitum. All rats were trained to voluntarily dive through a 100 × 60 cm Plexiglas underwater maze (∼5 m; see [McCulloch and Panneton 2003]) over the course of 8 weeks. Water was about 10 cm deep and was maintained at 30 ± 2°C. A raised platform placed in one corner of the tank was a finishing area where the rats exited the water. Rats were given ∼60 sec to explore this area after each training trial. During training, rats were held on a towel (30–60 sec) between each trial to let them get accustomed to human handling. Training was started by getting the rats to gradually swim the entire length of the maze. Once all rats completed the swim training, a Plexiglas chamber was placed in the opposite corner of tank from the finishing area. This chamber was designed in such a way that the only option for a rat to get out was to dive beneath the water surface. A Plexiglas cover was then placed just below the water surface to create an underwater tunnel. This tunnel was gradually extended with everyday training until the rats were able to swim underwater to the finishing area.

### Transmitter implantation

Once dive training was completed and all rats were over 300 g, a biotelemetric transmitter (model PA-C40; Data Sciences International [DSI], St. Paul, MN) was implanted following the surgical instructions provided by DSI. This transmitter was used to record pulsatile BPa from which HR was calculated. The rats were anesthetized with isoflurane (5%/vol initial induction, then 2–3%/vol maintenance). Under aseptic conditions, the tip of the transmitter catheter was inserted into the descending aorta, and the body of the transmitter was anchored in the peritoneal cavity using sterile nylon sutures. Ketoprofen (3–5 mg/kg sc) was given as a postsurgical analgesic. Rats were placed singly in cages under a heat lamp until they recovered from anesthetic. Supplemental ketoprofen (3–5 mg/kg sc) was given 24 h after surgery.

### Data collection and AEN surgery

One week after surgery, BPa was collected using a DSI receiver and decoder connected to a data acquisition program (Spike 2; Cambridge Electronic Design [CED], Cambridge, UK). Initially BPa was recorded in rats resting alone in their cages for 20 min, and then before, during, and after five voluntary dives. Data collection was repeated on the next day, for a total of 10 pre-AEN surgery dives. Following data collection, the transmitter-implanted rats were randomly assigned to two groups: (1) a Sham group that had sham lesioning of the AEN (*N* = 8) and (2) an Experimental group that had bilateral sectioning of AEN (*N* = 9). AEN sectioning procedure followed that of Rybka and McCulloch (2006). The rats were initially anesthetized using isoflurane (5%/vol initial induction, then 2–3%/vol maintenance) and then placed in a stereotaxic device (David Kopf Instruments, Tujunga, CA). Under aseptic condition, and after exposing the orbit and retracting the eyeball laterally, the AEN was identified and separated from the accompanying artery and vein. A 1-mm piece of nerve was removed from each AEN in the Experimental group, whereas the AEN was isolated but left intact in the Sham group. The same surgical procedures were repeated contralaterally. Ketoprofen (3–5 mg/kg sc) was used as a postsurgical analgesic. Following the veterinary advice and IACUC protocol, animals returned to diving after 7 days of surgical recovery. BPa was again collected in rats resting alone in their cages for 20 min, and then before, during, and after five voluntary dives. Data collection was repeated on the next day, for a total of 10 post-AEN surgery dives.

### Ammonia vapor and water stimulation of the nasal passages

This experiment evaluated whether the cardiovascular responses recorded during voluntary diving in the Experimental group after AEN sectioning had been cortically initiated. After the recording of all voluntary dives had been completed, and at least 9 days after the AEN surgery, some transmitter-implanted animals (Sham: *N* = 7; Experimental: *N* = 5) were anesthetized and had their nasal passages stimulated in an experiment similar to that performed by Rybka and McCulloch (2006). After the rats were anesthetized with isoflurane (5%/vol initial induction, then 2–3%/vol maintenance), a femoral vein was cannulated for drug administration. The trachea was transected and a tube was inserted caudally to enable breathing, and a nasopharyngeal tube was inserted rostrally to enable stimulation of the nasal passages (see below). The rats were then switched to urethane anesthesia (1 mg/kg, i.v.). BPa was recorded using the previously implanted DSI biotelemetric transmitter. The responses to two different methods of nasal stimulation were then recorded. First, a cotton swab soaked in 100% ammonia was held close to the external nares and a suction pump was connected to the nasopharyngeal tube to draw ammonia vapors through the nasal passages. Five ammonia vapor trials were recorded with 5-min intervals between each trial. Second, the nasal passages were stimulated with water by connecting a water-filled syringe to the nasopharyngeal tube and pushing ∼3 mL of room-temperature water through the nasal passages. Water was cleared from the nasal passages after each trial. Three water stimulation trials were recorded with 5-min intervals between each trial.

After the conclusion of all data collection, a gross postmortem analysis searched for the presence of the AEN, or any AEN nerve regeneration, within the orbits of Experimental rats.

### Statistical analysis

During voluntary diving trials, original pulsatile BPa traces were analyzed using Spike 2 to calculate mean BPa (in mmHg) and mean HR (in beats per min; bpm). The HR and BPa means of 10 pre-AEN surgery or post-AEN surgery dives were calculated for each rat, and those were averaged to create grand HR and BPa means (±standard errors [SE]) for each group. Cardiovascular data were analyzed using SigmaPlot 12 (Systat Software, San Jose, CA) with significance set at *P* < 0.05. For statistical analysis of cardiovascular data from voluntary diving rats, one- and two-way analysis of variances (ANOVAs) with repeated measures were used, as necessary, to determine whether the cardiovascular responses were different before and after the AEN had been sectioned. Data were also compared between the Sham and Experimental groups. For statistical analysis of cardiovascular data from anesthetized rats during nasal stimulation, one- and two-way ANOVAs with repeated measures were used, as necessary, to determine whether the cardiovascular responses were different between Sham and Experimental groups. When significant *P* values were encountered, post hoc testing with either Holm–Sidak's (for two-way ANOVAs) or Tukey's (for one-way ANOVAs) multiple pairwise comparison procedures determined which groups were significantly different. All figures were composed using SigmaPlot and CorelDRAW (Corel, Ottawa, ON, Canada).

## Results

### Behaviors of diving rats

Between each swim and dive trial, rats made no attempt to escape off the towel on which they were being held, and therefore appeared to have become accustomed to human handling. In the early training stages, rats gradually learned to swim a longer distance each day to the raised finishing platform. During the 1-min wait on the finishing area platform, some rats groomed themselves, explored the area, submerged their head underwater, and/or climbed off the platform and hopped onto the towel. When rats were placed in the starting chamber prior to a dive trial, defecation and teeth chattering were sometimes observed. During diving, rats took 12 ± 1 sec to swim the entire underwater distance. After completing underwater dives, rats often had a slightly bloody nose, especially after grooming themselves while waiting on the finishing area platform between trials.

### Voluntary diving: pre-AEN surgery

When rats were resting alone in their cages, Sham HR was 408 ± 11 bpm and BPa was 108 ± 3 mmHg. Experimental HR was 390 ± 16 bpm and BPa was 108 ± 2 mmHg. When being held on a towel between voluntary diving trials, Sham HR increased significantly by 29% to 526 ± 8 bpm from resting. Sham BPa also increased significantly by 18% to 127 ± 4 mmHg from resting. When being held on a towel between voluntary diving trials, Experimental HR increased significantly by 28% to 500 ± 14 bpm from resting. Experimental BPa increased significantly by 23% to 133 ± 2 mmHg from resting.

**Figure 1:**
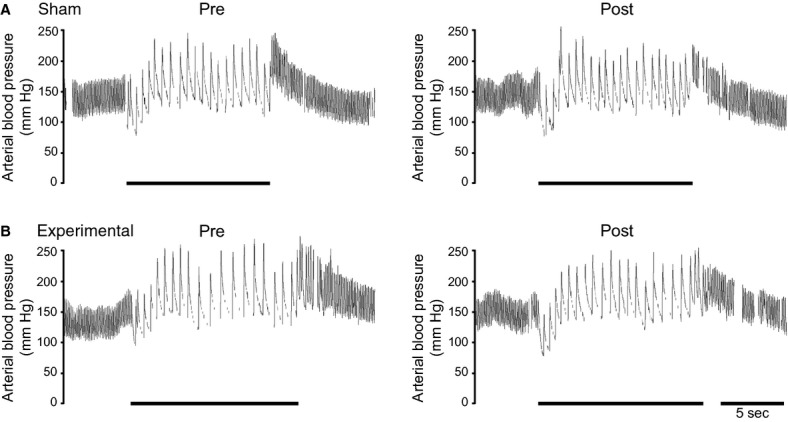
Original pulsatile arterial blood pressure traces in voluntarily diving (A) Sham and (B) Experimental rats. Pre and Post refers to before and after AEN surgery. In Sham rats, the AENs were isolated but were not cut. In Experimental rats, the AENs were cut bilaterally. Solid bars indicate periods of underwater submersion. Breaks in trace indicate periods when the radiotelemetric signal was lost.

**Figure 2:**
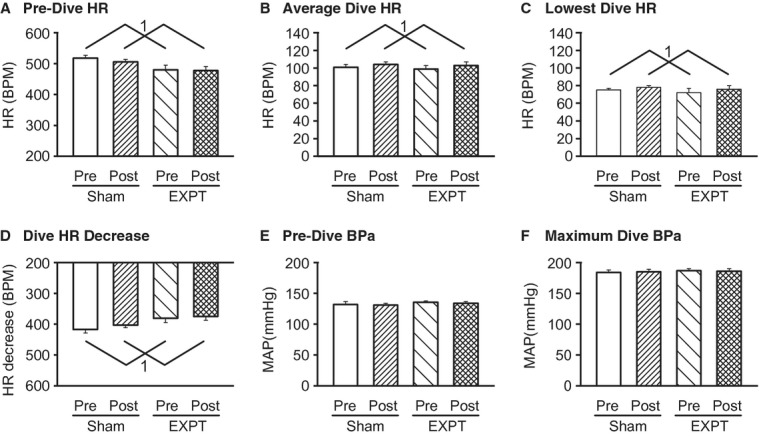
Mean HR and BPa (±SE) responses from voluntarily diving Sham (*N* = 8) and Experimental (EXPT; *N* = 9) rats both before (Pre) and after (Post) AEN surgery. (A) predive HR; (B) average HR during diving; (C) lowest HR during diving; (D) absolute decrease in HR during diving; (E) predive BPa; and (F) maximal BPa during diving. 1 = Pre-surgery HRs were significantly different from Postsurgery HRs as indicated by two-way repeated measures ANOVAs. The responses from Sham and Experimental rats were not significantly different from each other as indicated by two-way repeated measures ANOVAs.

Upon initiation of diving, HR decreased and BPa increased in both Sham and Experimental groups (Fig. 1A and B Pre). Sham HR decreased significantly by 81%, from 518 ± 9 to 101 ± 3 bpm, with the lowest HR reaching 75 ± 2 bpm (Fig. 2A–D). Sham BPa increased significantly by 40%, from 132 ± 5 to a maximum 184 ± 4 mmHg after a brief initial drop (Figs. 1A Pre, 2E–F). Experimental HR decreased significantly by 79%, from 480 ± 15 to 99 ± 5 bpm, with the lowest HR reaching 73 ± 5 bpm (Fig. 2A–D). Experimental BPa increased significantly by 37%, from 136 ± 2 to a maximum 187 ± 3 mmHg after a brief initial drop (Figs. 1B Pre, 2E–F).

Two-way ANOVAs with repeated measures indicated that prior to the separation into the Sham and Experimental groups, the major factor of AEN Surgery (Sham vs. Experimental) did not produce significantly different HR or BPa responses between the two groups either before or during diving. Therefore, prior to AEN surgery, HR and BPa from Sham and Experimental rats were not significantly different from each other.

### Voluntary diving: post-AEN surgery

When resting in their cages after recovering from the AEN surgeries, Sham HR was 383 ± 10 bpm and BPa was 108 ± 4 mmHg. Experimental HR was 378 ± 8 bpm and BPa was 117 ± 3 mmHg. When being held on a towel between voluntary diving trials, Sham HR increased significantly by 37% to 524 ± 8 bpm from resting. Sham BPa also increased significantly by 18% to 127 ± 3 mmHg from resting. When being held on a towel between voluntary diving trials, Experimental HR increased significantly by 32% to 497 ± 11 bpm from resting. Experimental BPa also increased significantly by 13% to 132 ± 2 mmHg from resting.

Upon initiation of diving, HR decreased and BPa increased in both Sham and Experimental groups (Fig. 1A and B Post). Sham HR decreased significantly by 80%, from 506 ± 8 to 104 ± 3 bpm, with the lowest HR reaching 78 ± 2 bpm (Fig. 2A–D). Sham BPa increased significantly by 42%, from 131 ± 3 to a maximum 185 ± 4 mmHg after a brief initial drop (Figs. 1A Post, 2E–F). Experimental HR decreased significantly by 78%, from 478 ± 13 to 103 ± 4 bpm, with the lowest HR reaching 76 ± 4 bpm (Fig. 2A–D). Experimental BPa increased significantly by 39%, from 134 ± 3 to a maximum 186 ± 4 mmHg after a brief initial drop (Figs. 1B Post, 2E–F).

Two-way ANOVAs with repeated measures indicated that after the AEN surgery the major factor of Surgery (Sham vs. Experimental) did not produce significantly different HR or BPa responses between the two groups either before or during diving. Therefore, 7 days after AEN surgery, HR and BPa from Sham and Experimental rats were not significantly different from each other, and the response to voluntary diving in the Experimental rats with bilateral AEN sectioning was no different from the response from Sham rats with intact AENs.

### Voluntary diving: pre- versus post-AEN surgery

Two-way ANOVAs with repeated measures used the main factors of Time (Pre-AEN Surgery vs. Post-AEN Surgery) and AEN Surgery (Sham vs. Experimental) to determine differences in HR and BPa during resting, holding, and diving. For all HR comparisons, there was no effect made by either the main factor of Surgery (Sham vs. Experimental) or main factor interaction (Time × Surgery). Therefore, during voluntary diving the HR responses from Sham and Experimental rats were not significantly different from each other. However, there was a significant effect made by the main factor of Time (Presurgery vs. Postsurgery) that affected both the Sham and Experimental rats similarly for predive HR (Fig. 2A), average dive HR (Fig. 2B), lowest dive HR (Fig. 2C), and decrease in HR during diving (Fig. 2D), but not resting HR or HR when being held on a towel.

For all BPa comparisons, there was no effect made by the main factor of AEN Surgery (Sham vs. Experimental). Therefore, during voluntary diving the BPa responses from Sham and Experimental rats were not significantly different from each other. However, for Resting BPa there was a significant effect of Time (Presurgery vs. Postsurgery), as well as a significant main factor interaction (Time × Surgery). For all other BPa comparisons, there were no main factor interactions (Time × Surgery). There was no effect of Time (Presurgery vs. Postsurgery) on predive BPa (Fig. 2E), maximum dive BPa (Fig. 2F), increase in dive BPa, or on BPa when being held on a towel.

### Nasopharyngeal response: ammonia vapor stimulation in anesthetized rats

**Figure 3:**
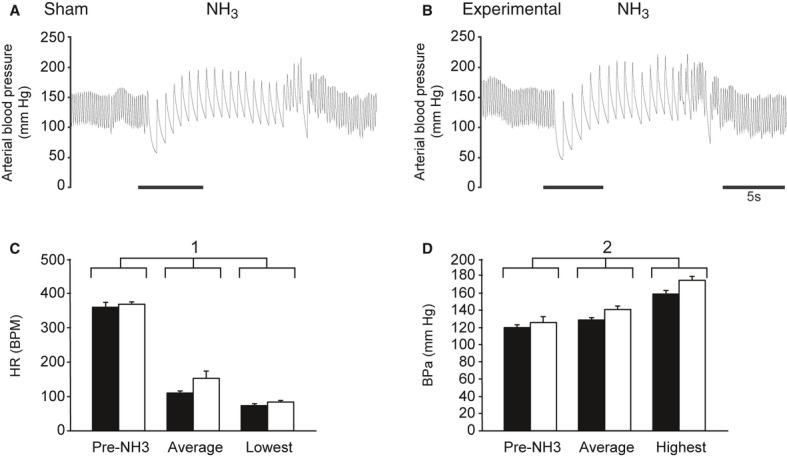
Original pulsatile arterial blood pressure traces in (A) Sham and (B) Experimental rats anesthetized with urethane during nasal stimulation with ammonia vapors. In Sham rats, the AENs were isolated but were not cut. In Experimental rats, the AENs were cut bilaterally. Solid bars indicate periods of nasal stimulation. (C) Mean HR (±SE) responses from Sham and Experimental rats before nasal stimulation (Pre-NH3), and the average and lowest HR during nasal stimulation. (D) Mean BPa (±SE) responses from Sham and Experimental rats before nasal stimulation (Pre-NH3), and the average and highest BPa during nasal stimulation. Filled bars = Sham; Open bars = Experimental. 1 = Pre-NH3, average and lowest HR were significantly different from each other, whereas the responses from Sham and Experimental rats were not significantly different from each other, as indicated by a two-way repeated measures ANOVA. 2 = Pre-NH3, average, and highest BPa were significantly different from each other, whereas the responses from Sham and Experimental rats were not significantly different from each other, as indicated by a two-way repeated measures ANOVA.

Similar to when they were diving voluntarily, all rats showed a decrease in HR and increase in BPa when their nasal passages were stimulated with ammonia vapors (Fig. 3). Sham HR decreased significantly from 359 ± 14 to 109 ± 6 bpm, with the lowest HR reaching 72 ± 6 bpm (Fig. 3A and C). Sham BPa increased significantly from 120 ± 3 to 128 ± 3 mmHg, with a maximum of 159 ± 4 mmHg (Fig. 3A and D). Experimental HR decreased significantly from 368 ± 7 to 153 ± 21 bpm, with the lowest HR reaching 83 ± 4 bpm (Fig. 3B and C). Experimental BPa increased significantly from 126 ± 7 to 141 ± 4 mmHg, with a maximum of 175 ± 4 mmHg (Fig. 3B and D).

Two-way ANOVAs with repeated measures indicated that after the AEN surgery neither the major factor of Surgery (Sham vs. Experimental) nor a main factor interaction (Stimulation × Surgery) produced significant differences between Sham and Experimental rats in the HR or BPa responses to nasal stimulation with ammonia vapors. During nasal stimulation, there was a major factor of Stimulation (preammonia, average, and lowest HR) that affected both the Sham and Experimental rats similarly for preammonia HR, average stimulation HR, and lowest stimulation HR (Fig. 3C). There was also a major factor of Stimulation (preammonia, average, and highest BPa) that affected both the Sham and Experimental rats similarly for preammonia BPa, average stimulation BPa, and highest stimulation BPa (Fig. 3D). Thus, HR and BPa responses from Sham and Experimental rats were not significantly different from each other during nasal stimulation with ammonia vapors.

### Nasopharyngeal response: water stimulation in anesthetized rats

**Figure 4:**
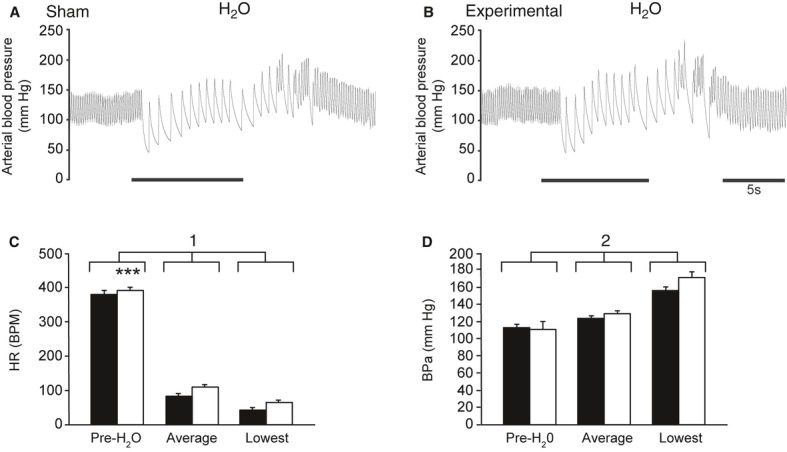
Original pulsatile arterial blood pressure traces in (A) Sham and (B) Experimental rats anesthetized with urethane during nasal stimulation with water. In Sham rats, the AENs were isolated but were not cut. In Experimental rats, the AENs were cut bilaterally. Solid bars indicate periods of nasal stimulation. (C) Mean HR (±SE) responses from Sham and Experimental rats before nasal stimulation (Pre-H_2_O), and the average and lowest HR during nasal stimulation. (D) Mean BPa (±SE) responses from Sham and Experimental rats before nasal stimulation (Pre-H_2_O), and the average and highest BPa during nasal stimulation. Filled bars = Sham; Open bars = Experimental. 1 = Pre-H_2_O, average and lowest HR were significantly different from each other, as indicated by a two-way repeated measures ANOVA. *= HR responses from Sham and Experimental rats were significantly different from each other, as indicated by the two-way repeated measures ANOVA. 2 = Pre-H_2_O, average, and highest BPa were significantly different from each other, whereas the responses from Sham and Experimental rats were not significantly different from each other, as indicated by a two-way repeated measures ANOVA.

Similar to when they were diving voluntarily, all rats showed a decrease in HR and increase in BPa when their nasal passages were stimulated with water (Fig. 4). Sham HR decreased significantly from 382 ± 10 to 83 ± 6 bpm, with the lowest HR reaching 43 ± 5 bpm (Fig. 4A and C). Sham BPa increased significantly from 113 ± 4 to 124 ± 3 mmHg, with a maximum of 156 ± 4 mmHg (Fig. 4A and D). Experimental HR decreased significantly from 392 ± 9 to 109 ± 7 bpm, with the lowest HR reaching 64 ± 6 bpm (Fig. 4B and C). Experimental BPa increased significantly from 112 ± 9 to 130 ± 3 mmHg, with a maximum of 172 ± 6 mmHg (Fig. 4B and D).

Two-way ANOVAs with repeated measures indicated that after the AEN surgery neither the major factor of Surgery (Sham vs. Experimental) nor a main factor interaction (Stimulation × Surgery) produced significant differences between Sham and Experimental rats in BPa responses to nasal stimulation with water. However, the major factor of Stimulation (pre-water, average, and highest BPa) affected both the Sham and Experimental rats similarly for prewater BPa, average stimulation BPa, and highest stimulation BPa (Fig. 4D). For HR, both the major factor of Surgery (Sham vs. Experimental) and the major factor of Stimulation (pre-water, average, and lowest HR), but not a main factor interaction, produced significant differences between the two groups in HR responses to nasal stimulation with water (Fig. 4C). Therefore, the HR responses from Sham and Experimental rats were significantly different from each other during nasal stimulation with ammonia vapors, although qualitatively the two groups produced similar responses (see Fig. 4C). Additionally, BPa responses from Sham and Experimental rats were not significantly different from each other during nasal stimulation with ammonia vapors.

Postmortem analyses of the orbits of Experimental rats confirmed that the AEN had been cut bilaterally, and found no evidence that the AEN had regenerated or that the two cut ends of the AEN had reattached.

## Discussion

Results from the present experiments indicate that during voluntary diving rats are able to initiate a full diving response in the absence of intact AENs. The voluntary diving responses from animals with intact AENs, which include an intense bradycardia and a 50+ mmHg increase in BPa, are similar to the responses from animals with bilaterally sectioned AENs. Additionally, the cardiovascular responses to nasal stimulation with either ammonia vapors or water in rats anesthetized with urethane are similar in animals with and without intact AENs. These findings are surprising, and contrary to our hypothesis, considering that previous studies have found the AEN to be essential for the full expression of the nasopharyngeal response (Rybka and McCulloch 2006). Because the cardiovascular response to nasal stimulation was still observed in anesthetized rats, it would seem unlikely that rats without intact AENs cortically, or consciously, initiated their diving response during voluntary diving. Therefore, the results from this study strongly suggest that other nerve(s) that innervate the nose and nasal passages are also able to provide an afferent signal sufficient to initiate a full diving response, although neuronal plasticity within the MDH may be necessary for this to occur.

Stimulation of nasal or other upper respiratory tract receptors are thought to initiate the cardiovascular responses observed in diving mammals (Butler and Jones 1997; McCulloch 2012). This has been found in seals (Dykes 1974), muskrats (Drummond and Jones 1979; Panneton 1991a,b), and rats (McCulloch and West 1992; McCulloch et al. 1995). The cardiovascular responses associated with voluntary diving in conscious rats are initiated upon submersion of the nares into the water, rather than just swimming on the surface of the water (McCulloch et al. 2010; Panneton et al. 2010). Additionally, infusing the nasal passages of anesthetized rats with the local anesthetic lidocaine eliminates the cardiovascular responses to nasal stimulation (McCulloch et al. 1995). Electrical stimulation of the AEN can produce a cardiorespiratory response similar to that of the diving response in both rats (Dutschmann and Herbert 1996, 1997, 1998) and muskrats (McCulloch et al. 1999). Likewise, bilateral AEN sectioning almost eliminates the bradycardia associated with nasal stimulation in anesthetized rats in acute experiments (Rybka and McCulloch 2006). Thus, the AEN, which innervates the external nares and internal nasal passages (Greene 1963), appears to be an important part of the afferent limb of this response. Additionally, the central projections of the AEN terminate in the ventral portions of the MDH (Panneton et al. 2006; Hollandsworth et al. 2009). This is the same brainstem location that contains secondary trigeminal neurons that are activated both during voluntary diving (McCulloch 2005) and nasal stimulation (Dutschmann et al. 1998; Rybka and McCulloch 2006; Hollandsworth et al. 2009). Also, unmyelinated fibers within the AEN may provide the afferent signal that causes initiation of the cardiovascular responses to nasal stimulation (Hollandsworth et al. 2009). Based on these previous findings, we had expected that the AEN would play an important, if not essential, role in initiating the diving response in voluntarily diving rats. Thus, we found results from the present experiments indicating that during voluntary diving rats are able to initiate a full diving response in the absence of intact AENs to be surprising.

The anatomy of the upper respiratory tract, from the nose to the nasopharynx to the larynx, is complex, and many reflexes can be generated through stimulation of receptors in these various locations (Widdicombe 1986; Widdicombe and Lee 2001). The defensive reflexes generated by these receptors are protective of the respiratory system in general, and the lungs in particular (Coleridge and Coleridge 1986). As would be expected, there is an inherent redundancy in the organization of the defensive reflexes, such that if a particular stimulus fails to initiate the appropriate defensive reflex, the next set of receptors in the airways could do so (Widdicombe and Lee 2001). Thus, in the context of the present experiments, if the AEN is nonoperational, it is reasonable to postulate the existence of other mechanisms that can initiate the protective apnea and other cardiovascular adjustments to conserve oxygen during underwater submergence.

In acute experiments, Rybka and McCulloch (2006) found that after bilateral AEN sectioning, the cardiorespiratory responses to nasal stimulation with ammonia vapors were severely attenuated, with almost no bradycardia. However, there was still a slight apnea and a significant 19% increase in BPa after nasal stimulation. Because an efferent response was observed in the absence of AEN afferent input, Rybka and McCulloch (2006) concluded that the nasal passages are innervated by nerve(s) other than the AEN that, when stimulated, can induce cardiovascular changes.

To determine the central projections of the nerves that innervate the nasal passages of rats, Anton and Peppel (1991) injected the transganglionic tracer wheat germ agglutinin conjugated to horseradish peroxidase (WGA-HRP) directly into the nasal passages. In contrast, Panneton (1991a), Panneton et al. (2006), and Hollandsworth et al. (2009) determined the central projections of the AEN by directly applying WGA-HRP to the AEN of rats. Anton and Peppel (1991) found a different pattern of afferent projections from the nasal passages, including a direct tracer projection to the nucleus tractus solitarius, compared to the studies that specifically injected only the AEN with WGA-HRP (Panneton 1991a; Panneton et al. 2006; Hollandsworth et al. 2009). Thus, Anton and Peppel (1991) results suggest that there are nerves other than AEN that innervate the nasal passage. This contention is supported anatomically, as the nasal passages and nasal mucosa of the rat are innervated by nerves from both the ophthalmic and maxillary divisions of the trigeminal nerve (Greene 1963).

Within the nasal passages of the rat, there is overlap of areas of innervation from different divisions, branches, and individual trigeminal nerves (Greene 1963). The nasociliary branch of the ophthalmic division divides into the ethmoidal (both anterior and posterior) and internal nasal nerves that innervate the nasal sinuses (ethmoidal and sphenoidal) and mucous membrane of the nasal cavity. The pterygopalatine branch of the maxillary division divides into three nerves (greater palatine, posterior superior nasal, and pharyngeal) that innervate the nasal cavity, nasal conchae, nasal septum, and nasopharynx. The anterior superior alveolar branch of the maxillary division divides into the nasal nerve that innervates the mucous membrane of the nasal cavity. It is possible that any of these nerves, in addition to the AEN, are capable of producing the afferent signal that can initiate the full cardiorespiratory responses to both voluntary diving and nasal stimulation. Indeed, it would be very interesting to determine if stimulation of any of these other nerves can produce the same cardiorespiratory responses as seen after stimulation of the AEN. However, in the rat the AEN is easily identified and accessed as it passes through the orbit. In contrast, the other trigeminal nerves that innervate the nasal passages travel mainly in the infraorbital nerve trunk in the infraorbital canal located at the base of the orbit. These nerves are therefore less easily identified or accessed as they branch to innervate the nose and nasal passages. Thus, investigation of the physiological responses to stimulation of these other nerves would be surgically more complicated than is investigation of the AEN.

In addition to nervous innervation of the upper respiratory tract, the cardiorespiratory changes observed during voluntary diving in rats after bilateral AEN sectioning could have been produced through cortical initiation. In anticipation of submergence the rats could have cortically initiated cardiorespiratory changes to conserve oxygen while under water. We investigated this possibility by anesthetizing the same rats with urethane to eliminate the possibility of conscious initiation of cardiorespiratory reflexes. As stimulating the nasal passages with either ammonia vapors or water still produced a full cardiorespiratory response in anesthetized rats, we concluded that cortical involvement was not required for initiation of this response. This then suggests that the diving response observed during voluntary diving in the rats with bilateral AEN sectioning was also not cortically initiated. Instead it reinforces an alternative hypothesis that stimulation of other nerve(s) in the upper respiratory tract initiated the responses to both nasal stimulation and voluntary diving in these animals.

There is a major discrepancy between the results from this study and those from (Rybka and McCulloch 2006). The present experiments found that the cardiovascular responses to both voluntary diving and nasal stimulation were similar regardless of whether the animals had intact AENs or bilaterally sectioned AENs. Contrary to these results, Rybka and McCulloch (2006) found that the cardiac responses to nasal stimulation with ammonia vapors were severely attenuated in animals after bilateral AEN sectioning. A notable procedural difference between this study and the study by Rybka and McCulloch (2006) is that in this study we waited at least 9 days after cutting the AEN before the responses to nasal stimulation under anesthesia were recorded. The study by Rybka and McCulloch (2006) was acute, and the cardiorespiratory responses to nasal stimulation were recorded within hours after bilateral AEN sectioning. The waiting period in this study followed veterinary advice to provide time for surgical recovery before allowing the rats to return to swimming and diving. This procedural difference could provide an explanation as to why the full cardiovascular reflex responses to both voluntary diving and nasal stimulation are present after bilateral AEN sectioning in this study. It is possible that during the intervening 7-day period either (1) the AENs have regenerated after being cut, or (2) other nerve(s) have taken over the function of AEN to provide the appropriate afferent signal to initiate the cardiorespiratory responses.

### AENs have regenerated

This possibility seems unlikely to explain the full diving response shown by the Experimental rats after bilateral AEN sectioning. Because a 1 mm piece of the nerve was removed at the time of AEN sectioning, it is unlikely that the nerve had regenerated in a mere 7-day period in all the rats. Also, at the time of the AEN surgery it was noticed that both ends of the cut nerve retracted further away from each other after the sectioning. Moreover, Rybka and McCulloch (2006) showed that cutting the AEN unilaterally, reducing 50% of the full AEN afferent signal, resulted in only a partially attenuated nasopharyngeal response. Even if few fibers managed to grow back and recover function, it cannot explain why the full and complete diving and nasopharyngeal responses were observed in our study. Furthermore, at the time of postmortem inspection of the orbit after completion of data collection, we did not observe any sign of AEN regeneration. Due to all of these observations, we are convinced that the AENs did not regenerate during the time course of this study.

### Other nerves have taken over the function of AEN

As discussed above, there are other nerves besides the AEN that innervate the nares and nasal passages. Results from this study suggest that one or more of these afferent nerves are indeed capable of producing the afferent signal that can initiate the full cardiovascular responses to both voluntary diving and nasal stimulation. However, the cardiorespiratory responses to nasal stimulation are diminished immediately after bilateral AEN sectioning (Rybka and McCulloch 2006) but fully restored by at least 9 days later (present study). Thus, whatever this alternate neural pathway may be, a few days are required for it to become fully operational. It seems unlikely that it would take a few days for non-AEN upper airway receptors to be able to signal the presence of either water or ammonia vapors in the nasal passages. Rather, if non-AEN sensory receptors are stimulated, they should be stimulated regardless of whether the AEN is intact or not. Instead, it is more likely that the delay in being able to activate the cardiorespiratory responses after nasal stimulation is due to neuronal rewiring within the MDH.

Secondary neurons within the MDH normally receive AEN afferent information (Hollandsworth et al. 2009). However, after bilateral AEN sectioning the secondary MDH neurons would no longer receive this AEN afferent input. It may be necessary for the non-AEN nerve(s) to develop new terminal projections within the Central Nervous System and create synaptic connections with these secondary MDH neurons. Alternatively, there may be previously existing but functionally weak (i.e., “silent”; see [Holland 1996; Navarro et al. 2007]) synapses between the non-AEN nerve(s) and secondary MDH neurons that strengthen and increase efficacy after AEN sectioning (Devor and Wall 1981; Waite 1984; Navarro et al. 2007). Once these synaptic connections are made, or perhaps strengthened, afferent information from the nasal passages could reach the MDH neurons. This neuronal plasticity may take a few days, which would account for (1) the delay in the full return of the cardiorespiratory responses to nasal stimulation with ammonia vapors, and (2) the observation of a “normal” diving response during voluntary diving 7 days after the AEN sectioning. However, while allowing time for neuronal plasticity to occur may be a plausible explanation for the delay in the return of the reflex responses, it does seem counterintuitive that a “back-up” defensive reflex takes as long as 7 days to become operational. A protective and defensive “back-up” mechanism should immediately be fully operational in case the primary mechanism is not functional, as would have happened after the AENs had been sectioned bilaterally. Even so, the delay encountered in restoring this autonomic reflex is much faster (within 7 days) than trigeminal somatosensory plasticity, which in adult rats may take substantially longer (∼60 days; [Klein 1991; Renehan et al. 1989]) or perhaps may not even occur (Waite 1984). This potentially may highlight a difference within the trigeminal system between plasticity of autonomic reflexes versus that of the somatosensory system.

In conclusion, we found that 1 week after being cut bilaterally the AENs are not essential for initiating the diving response in voluntarily diving rats. It is not surprising that there are redundant neural pathway(s) that can initiate this response, as the role of the diving response is to initiate apnea and protect the animal from developing hypoxia during underwater submergence. What is surprising, however, is that this redundant defensive mechanism apparently takes at least a few days to become fully operational. Further study is needed to determine what this “back-up” system is and define its characteristics during initiation of this reflex response.
